# God in body and space: Investigating the sensorimotor grounding of abstract concepts

**DOI:** 10.3389/fpsyg.2022.972193

**Published:** 2022-11-03

**Authors:** Suesan MacRae, Brian Duffels, Annie Duchesne, Paul D. Siakaluk, Heath E. Matheson

**Affiliations:** Department of Psychology, University of Northern British Columbia, Prince George, BC, Canada

**Keywords:** grounded cognition, embodied cognition, concepts, abstract concepts, Representation

## Abstract

Abstract concepts are defined as concepts that cannot be experienced directly through the sensorimotor modalities. Explaining our understanding of such concepts poses a challenge to neurocognitive models of knowledge. One account of how these concepts come to be represented is that sensorimotor representations of grounded experiences are reactivated in a way that is constitutive of the abstract concept. In the present experiment, we investigated how sensorimotor information might constitute GOD-related concepts, and whether a person’s self-reported religiosity modulated this grounding. To do so, we manipulated both the state of the body (i.e., kneeling vs. sitting) and the state of stimuli (i.e., spatial position on the screen) in two tasks that required conceptual categorization of abstract words. Linear Mixed Effects model fitting procedures were used to determine which manipulated factors best predicted response latency and accuracy in both tasks. We successfully replicated previous research demonstrating faster categorization of GOD-related words when they were presented at the top of the screen. Importantly, results demonstrated that the kneeling posture manipulation enhanced this effect, as did religiosity, as participants who scored higher in religiosity showed a greater effect of the posture manipulation on the speed with which word categorization occurred when those words were presented in the higher visuospatial presentation condition. Overall, we interpreted our findings to suggest that directly manipulating sensorimotor information can facilitate the categorization of abstract concepts, supporting the notion that this information in part constitutes the representation of abstract concepts.

## Introduction

The ability to recognize and identify people, places, and things in the environment is essential for successfully interacting with the world. For example, when we see a hammer we know immediately what that object is and what it is used for without examining it in detail. How is this identification possible? The framework of grounded cognition posits that our understanding of objects in the environment is at least partially based on sensorimotor information that is obtained from our past experiences with objects (e.g., Barsalou, 1999, 2008). According to this proposal, when we interact with an object, we encode sensorimotor information (including action-oriented information), visual information, and auditory information (among other types of information gained through bodily experience) that are activated simultaneously in lived experience. This correlated activity becomes associated so that, in the future, object identification causes some or all associated regions to become active (Hebb, 1949; see Pulvermuller, 1996). This idea is captured in a model by Meyer and Damasio (2009), who proposed a neural account of this type of sensorimotor grounding. In this model, co-activation patterns across the modalities is captured by higher level regions called convergence divergence zones (CDZs). The distributed pattern of activity can be elicited when sensory information is experienced (i.e., in encoding), and to activate sensorimotor information in reverse (i.e., during identification). Critically, this model reflects known neurobiological constraints; it is organized hierarchically, it incorporates reciprocal connectivity, and it functions using Hebbian associations. Overall, this model, and similar ‘embodied’ or ‘grounded’ models (Pulvermuller, 1996; Hommel, 2015; Fernandino et al., 2016; Matheson and Barsalou, 2018, for a review) suggests that the representation of people, places, and things is a distributed pattern of associated activity across the cortex that is constrained by our bodily interactions in the environment. Importantly, in these models, we can interpret this pattern of re-activation as a *neural representation* of the person, place, or thing; it is not simply ‘related to’ or ‘associated with’ the representation of the object but rather the representation of the object is constituted by this activity (see Shapiro, 2019). To understand a concept then means to reactivate distributed, hierarchically organized, bodily constrained, sensorimotor information.

There is behavioural support for models that posit modality-related reactivations like those described in the CDZ model. For instance, Zwaan et al. (2002) presented participants with sentences that implied particular shape information about an object that was described in the sentence. The authors then presented a picture of the object in a way that was either consistent or inconsistent with the implied shape. For instance, one sentence was “the eagle flew over the nest.” The picture following that sentence was either an eagle flying with its wings spread (i.e., congruent with the shape implied by the sentence) or an eagle perched in a nest with folded wings (i.e., incongruent with the shape implied by the sentence). Participants were instructed to decide if the object presented was the one described in the sentence. Critically, in both cases, pictorial representations of the same concept (i.e., EAGLE) were presented; of interest was the difference in response latency for images of the object with the congruent shape compared to the incongruent shape. The authors reported that it took participants longer to categorize the object when it was presented in a posture incongruent with the shape implied by the sentence. This is a surprising finding because if the shape of a presented object was irrelevant to activating its conceptual representation, there should be no difference between the response latencies of the two conditions. This result is consistent with the notion that, to interpret the sentence, participants were relying on a reactivation of visual information of the object that is specific to the context (Zwaan and Pecher, 2012 and Zwaan et al., 2018, for replications of this robust effect).

Importantly, this type of finding suggests that visual information is active during conceptual processing, but it does not tell us about its functional role or whether representations of concepts are constituted by it (see Matheson et al., 2014, for a critique). To test for the functional role of sensorimotor information, concurrent tasks that affect specific modalities must be used (Ostarek and Bottini, 2021). Activation of the specific sensorimotor regions that are constitutive to concept representation should change concept representation. The directionality of the predicted change to concept representation has been ambiguous in the literature with some studies reporting that the effects are inhibitory (for example Yee et al., 2013) while others have found a faciliatory effect (for example Matheson et al., 2019). For instance, Yee et al. (2013) had participants do a three-part clapping pattern (i.e., paddy-cake) while identifying whether words were concrete or abstract. The words varied in the degree to they were experienced through haptic interactions with the objects. The authors reasoned that, if understanding words like ‘hammer’ requires a partial re-activation of motor information associated with HAMMER, the dual task should interfere with that activity and impair categorization. The results suggested that the secondary task of clapping did indeed impair the ability to categorize words that were primarily experienced through touch. This finding suggests that motor information, activated by the clapping task, was not available to be used in a simulation that was constitutive of the conceptual judgment (see also Paulus et al., 2009; Yee et al., 2013; Matheson et al., 2019). Matheson et al. (2019) applied similar reasoning to the development of their experimental paradigm, however the effects they reported were faciliatory. Matheson et al. (2019), trained participants to use novel tools. Participants then performed familiarity judgements about tool names during a simultaneous motor task. Matheson et al. (2019) reported a faciliatory effect of the motor task where participants who were trained to use the tools identified them faster while performing the motor task. Though the issue of directionality needs further elucidation (See discussion in: Pecher et al., 2021), these findings suggest that activated motor information has a functional role in conceptualization.

Hammers and eagles are concrete objects. That is, they can be experienced directly though the body and actions of the body. Though we have reviewed evidence above of sensorimotor grounding for concrete objects, a common criticism of grounded cognition is that it does not explain how *abstract* concepts are represented (e.g., Dove, 2011; see Mahon and Hickok, 2016). That is, how do models like the CDZ model explain the representation of things that cannot be experienced in any direct way through sensorimotor modalities, such as JUSTICE, DEMOCRACY, or FAITH? Because, by definition, abstract concepts are intangible, it is difficult to account for how they could be understood through the reactivation of sensorimotor representations derived from bodily experience (Bolognesi and Steen, 2018). Though this criticism does appear to challenge embodied and grounded models of conceptual processing, there are a number of responses to it (Reilly et al., 2016; Borghi et al., 2017; Dove, 2021). First, some authors suggest abstract concepts are not static entities that exist in the mind/brain and are activated in isolation, but rather may be experienced and activated across a diversity of contexts, to support situated action (Barsalou, 2016; Yee and Thompson-Schill, 2016). Thus, the concepts of JUSTICE, DEMOCRACY, or FAITH are supported by the activation of all the sensorimotor information that is active across a variety of contexts, including information about typical actions associated with these concepts, as well aspects of emotion and motivation (see Newcombe et al., 2012), and the way we use language socially (Borghi et al., 2013; Barsalou et al., 2018). Thus, abstract concepts may be supported by the activation of a wide variety of situated sensorimotor information and, importantly, its integration through co-occurrence. Overall, there is no need to posit a type of symbolic representation that is cleaved from sensorimotor experience.

One prominent version of this approach is Conceptual Metaphor Theory (Lakoff and Johnson, 1980) developed within cognitive linguistics. This theory suggests that abstract concepts can *only be* understood by mapping aspects of sensorimotor experience onto our abstract concepts; that is, we understand abstraction through a type of sensorimotor metaphor. For example, when we describe the dissolution of a social relationship we use phrases like “headed in different directions,” “at a crossroads,” or “hit a rough patch.” This type of linguistic analysis shows that we at least partially understand the abstract concept of a relationship by mapping our physical experience associated with travelling—which is directly experienced in sensorimotor modalities, into the abstract domain of friendship (see Lakoff and Johnson, 1999, for extensive analysis). Though this approach is not without limitations (Kovecses, 2008), and some theorists argue that the concrete/abstract distinction is illusory and distracting (Barsalou et al., 2018), it nonetheless provides a robust account of how abstract concepts, like concrete ones, are grounded, in one way or another, in sensorimotor experiences (Johnson, 2017, for an analysis of this hypothesis).

Religious concepts, such as GOD and DEVIL, are some of the most meaningful to many people and are of great significance in many parts of the world. Religious concepts are widespread and consistent across members of cultural and religious groups. People *do* have sensorimotor experiences associated with these concepts; experiences of using language, viewing art, and performing ritual. However, these words refer to things that are not literally experienced directly through our sensorimotor modalities; that is, people do not routinely touch, see, hear, and smell the things the words refer to. Thus, according to the grounded framework presented here, these types of concepts are grounded in sensorimotor experiences that unfold in situational contexts related to them (see Barsalou et al., 2005 for extensive discussion).

Given the widespread adoption of ritual and the widespread representations of faith concepts in media, grounding may occur *via* the motor system (e.g., representations of the body’s configuration or action during religious ritual) and the visual system (e.g., representations of visual depictions of words and their referents) (Soliman et al., 2015). A small body of research has explored this issue. For instance, a seminal study tested the association between GOD-related words and visual space using a categorization task (Meier et al., 2007). Participants in this study sorted words presented on a computer monitor into one of two categories: GOD-related words or DEVIL-related words. Critically, the words were presented at the top of the screen or the bottom of the screen. The authors reasoned that depictions of God as being higher up or ‘on high’ (and conversely that the devil is below us) are not arbitrary but reflect a visuospatial grounding of the concept. The results showed that participants were faster to categorize GOD-related words when they were presented at the top of the screen. One interpretation of this finding is that the activation of the visual “up-ness” in vertical space facilitates categorizing the GOD-related words because the representation of the words is partially constituted by re-activations of ‘up-ness’ across instances of the GOD concept.

Subsequent studies have used GOD-related words to bias attention towards different spatial quadrants of a computer screen (Chasteen et al., 2010). In these studies, following the presentation of a word relating to GOD, DEVIL, or a neutral word, a dot appeared in a random position on the screen. If the word presented was related to GOD or DEVIL, participants responded by pressing a key; if the word was neutral they were told not to respond when they saw the dot. When participants read GOD-related words they were faster to react to dots presented higher and to the right of the screen; when they read DEVIL-related words they responded faster to dots in the lower and the left quadrant. One interpretation of these findings is that to make the judgments participants activated sensorimotor information related to visuospatial experience, and the activation of this visuospatial information primed or facilitated responding to the dots when they fell in the primed location. This again suggests that there is an association between GOD and UP.^1^ There are also associations reported between DEVIL and DOWN however these effects are less robust (Meier et al., 2007).

These studies suggest that the sensorimotor experience of ‘up-ness’ becomes constitutive of the GOD concept, that is, it has become part of the distributed, modality-related re-activation that allows us to understand and use the concept of GOD. This is consistent with how people encounter and generally experience GOD-related concepts. That is, the concept is often represented ‘on high’ in language, or literally higher in visual depictions (e.g., paintings), and people often engage in rituals that place GOD, experientially, in the sky (e.g., when singing to the heavens). These contexts ensure the co-activation of up-ness and other related sensorimotor features associated with GOD, and therefore can serve to partially ground this abstract concept in sensorimotor experience. However, it is unlikely that vertical space is the only or even primary experience with which the concept of GOD is developed. Indeed, religious contexts and rituals are filled with consistent bodily practices that would serve to associate co-activations of modality-related information. For instance, Barsalou et al. (2005) suggest that supplication is part of the experience of religious ritual for many of the world’s religions. Kneeling, in general, communicates submission (e.g., kneeling before a king), and the experience is extended when people kneel to pray, or otherwise supplicating before a religious deity. Thus, in addition to vertical space, bodily information related to kneeling (i.e., the muscle configurations used to execute the action; proprioceptive information associated with the arrangement of the limbs; somatosensory information from feedback in the legs and floor, etc.) will also often be co-activated in religious contexts. Kneeling, therefore, may be another way in which GOD is grounded in bodily experience.

This idea has yet to receive any direct experimental attention. In a related study, Fuller and Montgomery (2015) had participants assume either low constricted postures or high expansive postures and then complete surveys on religious ideas. They found that, for participants who identify as religious, the brief low body posture manipulation lead to higher religiosity scores than those in the high body posture condition. This study shows that posture might temporarily influence self-reported religious belief, but does not directly assess whether body posture functionally contributes to understanding and representation of abstract GOD-related concepts.

**Figure 1:**
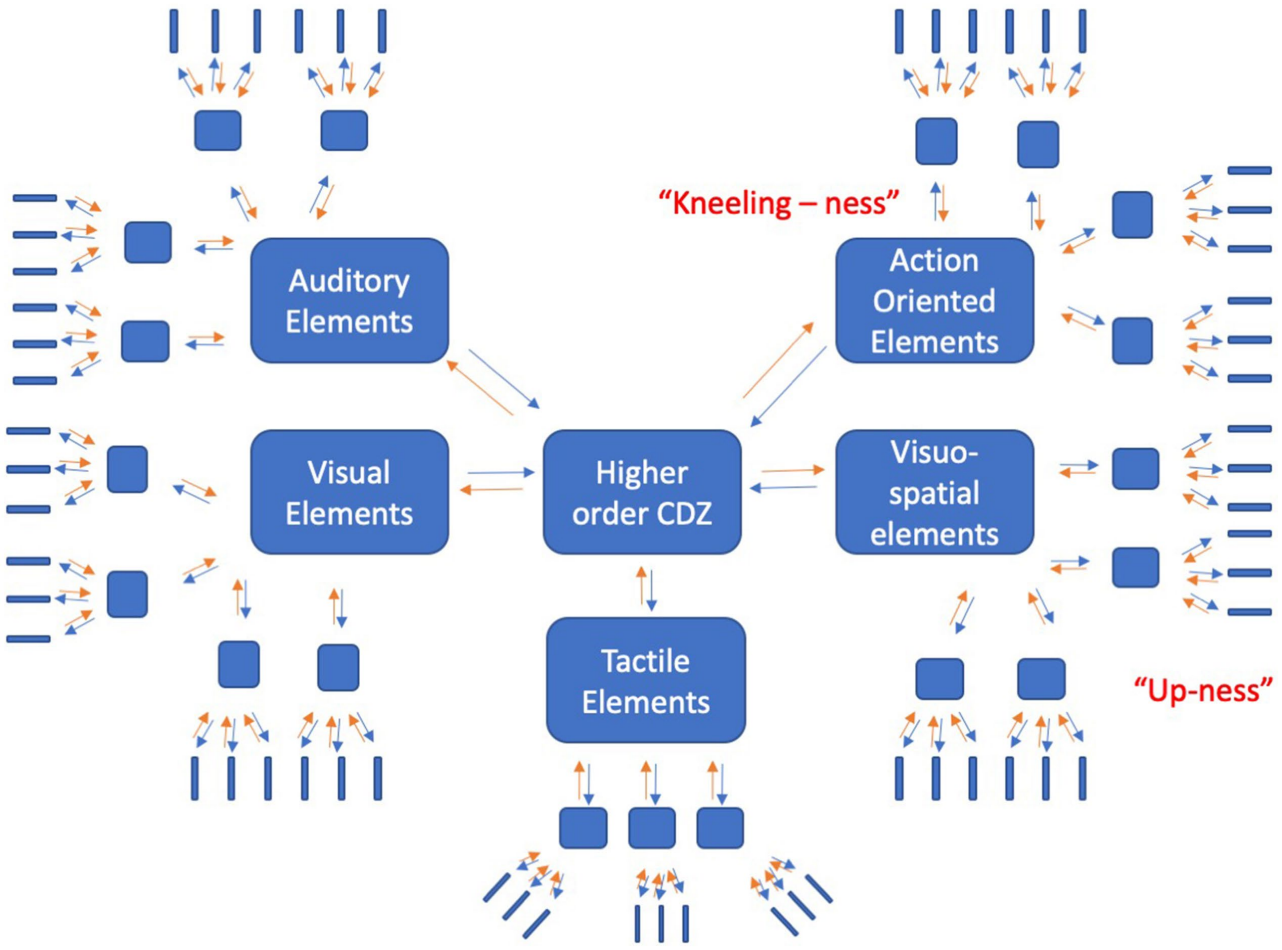
Conceptual representation of GOD-related concepts. Note: The sensorimotor modalities are organized hierarchically, with lower levels encoding low-level information (blue elongated rectangles) and higher convergence zones encoding co-activations within the modalities (blue squares), and even higher convergence zones encoding co-activations across different modalities (large blue rectangles). Activation can spread from the lower level regions upwards (orange arrows) and from higher order convergence zones to lower level regions (blue arrows). In representing GOD-related concepts, action-oriented elements (kneeling-ness) and visuospatial elements (up-ness) are constitutive and therefore important to the representation. Adapted from Meyer and Damasio (2009).

If the experience of GOD is often connected with the perceptual experience of vertical space *and* the bodily state of a supplicative posture, then these experiences can be used to ground GOD-related concepts. In the present study, we used a categorization task (CAT) and a version of the implicit association task (IAT) modified from Meier et al. (2007) to test this idea. We used these two tasks because they have shown replicability in the literature in probing abstract concepts, and because we seek to provide convergent and complimentary evidence for the role of body, space, and related religious experience on abstract conceptual representation. We reasoned that bodily posture should interact with vertical space in modulating conceptual performance on GOD-related words, by virtue of either priming or interfering the activation of sensorimotor information that is constitutive of GOD-related concepts. As per the CDZ model, we expect that there are many sources of information that are constitutive of a representation of these concepts. We suspect that these components of the representation are distributed across sensorimotor modalities. Built on principles of Hebbian learning, we assume that activation in one modal region can induce activity across the entire representation and therefore, we hypothesize that activating aspects of GOD-related representations (e.g., kneeling-ness, up-ness) will serve to prime GOD-related concept recognition, thus leading to faster and potentially more accurate categorization (See Figure 1).

Importantly, we measured participant religiosity to assess whether the effects of kneeling or visuospatial position on categorization of GOD-related words changes with individual religious experience; that is, one can reason that the more that a person experiences visual depictions of religious concepts and supplicates before them, the more these variables should ground the concept.

## Materials and methods

### Participants

For this study, we collected data from 63 undergraduate students from the University of Northern British Columbia. The average age of participants was 20.78 (*SD* = 2.86). In the CAT, 57 students completed the task; in the IAT 56 participants completed the task. Participants were convenience sampled from the department’s subject pool and participants were compensated with credit towards coursework. Participants provided written consent and ethics was approved by the Research Ethics Board at the University of Northern British Columbia.

### Materials and design

Participants performed the IAT, CAT, and completed a number of questionnaires. The IAT, the CAT, and the survey questions were programed using Direct RT (Version 2014, Empirisoft Corporation). The tasks were displayed on a 24-inch LED monitor (Dell P2417H with a resolution of 1920 × 1,080 and a refresh rate of 60 Hz). Stimuli were presented in 22-point Arial font. The font colour was white and appeared on a black background. During the IAT the target word for each trial was presented in the center of the screen. For the CAT, the words were presented in one of two vertical positions: either at 748 pixels from the top of the monitor or 20 pixels from the top of the monitor. In addition, for both tasks, the presentation of words was grouped by category; that is, the GOD-related and DEVIL-related words were presented together, the POWERFUL-related and POWERLESS-related words were presented together, and the SKY-related and OCEAN-related words were presented together. The order in which participants categorized these three sets of words was randomized between participants.

The complete set of stimuli used in the two tasks are shown in Table 1. We used the same stimulus set as used by Meier et al. (2007) with minimal changes. The words varied in their association to either GOD or DEVIL. In the CAT we compared religious words to words that related to social power and to landscapes. Ocean and sky words were selected from a pool of ocean and sky words used in Pecher et al. (2010). The power words were selected from Schubert (2005). The order in which participants categorized GOD-DEVIL, POWERFUL-POWERLESS, or OCEAN-SKY words was randomized between participants.

**Table 1:** Religious word concepts taken from Meier et al., 2007 and non-religious concepts taken from Pecher et al., 2010 (OCEAN and SKY words) and Schubert, 2005 (POWERFUL and POWERLESS words).

GOD words	DEVIL words	POWERLESS words	POWERFUL words	OCEAN words	SKY words
Almighty	Antichrist	Apprentice	President	Anchor	Helicopter
Creator	Demon	Assistant	Boss	Submarine	Balloon
Deity	Lucifer	Student	Professor	Shell	Kite
Lord	Devil^*^	Defendant	King	Whale	Eagle

* We used ‘Devil’ rather than the word ‘Satan’ from Meier et al. (2007) in an attempt to use a more general word.

When they arrived in the lab participants were shown two different postures, a keeling posture and a sitting posture. We used a cover story to suggest to the participants that we were interested in ergonomics. For the sitting posture, participants were directed to sit on a chair positioned in front of the monitor. For each participant, the monitor was adjusted to ensure that it was centered and at eye level. Participants were reminded to keep both feet flat on the floor and keep their back straight in an ergonomically correct position. For the kneeling condition the chair was moved behind the participants and they were given a yoga matt kneel on, shins parallel to the floor and their feet behind them. Participants were directed to kneel in front of the computer and were reminded to keep their backs straight and maintain an ergonomically correct position. For each participant, the monitor was adjusted if necessary to ensure it remained centered and at eye level. Participants were given breaks between each block to ensure that they were comfortable during the experiment.

The order in which the participants completed the IAT and the CAT task was counterbalanced so that half of the participants saw the IAT first and half of the participants saw the CAT first. The participant remained in the sitting or kneeling posture to complete both the CAT and the IAT. They then assumed the other posture and completed the IAT and CAT again.

While completing the tasks, participants were told to keep their index fingers of each hand on the response keys (Q and P) for the entire time during the IAT and the CAT. They were instructed to respond as quickly and accurately as possible and were told some mistakes were acceptable provided they responded as fast as possible. Participants always completed the religiosity inventory last and were seated to complete this inventory.

### IAT procedure

Participants completed an IAT with GOD-related and DEVIL-related words and vertical direction words. The IAT contained 7 blocks of trials (where a block is a short series of trials). On each trial, a word appeared on the screen (e.g., god, devil, up, down) and the participant categorized the word. To categorize the word, participants needed to determine if the word presented in the center of the screen best matched the category shown on the top left corner or the top right corner of the screen. They then indicated these choices by pressing either the Q button with their left hand or the P button with their right hand. Response latency and accuracy were recorded. In the first block participants categorized words as either belonging to the category DEVIL or to the category GOD. In the second block they categorized words as relating to UP or DOWN. The third block was a practice block where participants categorized words as belonging to a DEVIL/UP or GOD/DOWN category. The fourth block was a continuation of the third block, where participants continued to make the same categorizations but now their responses were recorded for analysis. In the fifth block participants were retrained to use the opposite buttons for GOD and DEVIL. In the sixth block, they practiced categorizing words into a DEVIL/DOWN or GOD/UP category. In the final block (the seventh block) they continued to make these categorizations, but their responses were recorded for analysis. Order of the fourth block and the seventh block was counterbalanced (along with the preceding practice block) so that half of the participants saw GOD and UP together first and half of the participants saw GOD and DOWN first. This was done to control for order effects.

In each trial a blank screen was presented for 150 ms, followed by a stimulus word in the centre of the screen. Participants responded to the word by pressing either P with the right hand or Q with the left hand. The responses were counterbalanced across participants. While participants were making categorization responses, headings appeared at the top of the screen to indicate the category of words that were being sorted (i.e., GOD-related and DEVIL-related words, GOD-related and UP-related words, DEVIL-related and DOWN-related words, etc.). A black screen appeared following each response for 150 ms. Following the methods of Meier et al. (2007), when participants made an error the word INCORRECT was presented on the screen in red font and remained on the screen for 1,500 ms before returning to the task. The use of feedback was intended to motivate fast and accurate responding.

### Categorization task procedure

In this task, participants categorized words as either belonging to one of three categories: GOD/DEVIL, OCEAN/SKY, or POWERFUL/POWERLESS. The GOD/DEVIL categorization was a replication of Meier et al. (2007). The two other conditions varied in concreteness, and were both more concrete than the GOD/DEVIL category. We had participants judge both concrete words relating to the OCEAN or SKY (e.g., anchor vs. helicopter) and words of an intermediate level of abstractness relating to levels of social power; namely, POWERFUL vs. POWERLESS (e.g., boss vs. worker).

Each word was proceeded by a circle fixation at the centre of the screen. The fixation remained visible for 300 ms, followed by a blank screen that also appeared for 300 ms. The word then appeared in either the top position (20 pixels from the top of screen) or in the bottom position (748 pixels from the top of the screen). Again, participants categorized the words by pressing the P or Q keys (counterbalanced across participants). Correct responses were followed by a blank screen for 300 ms. Incorrect responses were followed by the word INCORRECT (in red font), which remained on the monitor for 1,500 ms, after which trials resumed.

### Questionnaire procedure

Participants completed three questionnaires measuring religiosity. These questionnaires were completed after the IAT and CAT and were completed in a sitting position in front of the computer monitor. Participants only completed the religiosity questionnaires once. First, participants completed the 20-Item Dimensions of Religiosity questionnaire (Joseph and Diduca, 2007). In this questionnaire, participants used a 5-point Likert scale to rate the extent to which they endorsed statements concerning their preoccupation, conviction, emotional involvement, and the extent to which they seek guidance from their religion. Participants used a similar 5-point Likert scale to complete the 10-item Religious Commitment Inventory (Worthington et al., 2003). Finally, participants completed the Nearness to God inventory (Gorsuch and Smith, 1983) which is a derivation of the Religious Attitudes Inventory (Broen, 1957). In this questionnaire participants traditionally indicate whether they agree or disagree with 6 statements; in the present study we used a 5-point Likert scale instead for consistency of use with the other subscales. Meier et al. (2007) report a similar transformation of the subscale, using a four-point scale rather than the dichotomous subscale in the original publication (Gorsuch and Smith, 1983). To construct a composite score assessing the overall religiosity of participants in this study, the scores were averaged for each inventory and then those averages were averaged. This composite religiosity score was used as the index of the participants religiosity.

The order in which the participants completed each of the surveys was randomized for each participant. In each survey some items were reverse scored to ensure that participants were reading the questions and considering their responses.

### Analytical strategy

#### Linear mixed effects models

Linear Mixed Effects models (LME) models are able to circumvent limitations of ANOVA (Brown, 2021). First, LME is a type of regression able to readily deal with hierarchical (i.e., non-independent) data by setting random intercepts which account for the differences of individual participant performance. Second, ANOVA necessarily loses information by aggregating information either across individual or across stimuli; data is not aggregated in this way in LME procedures. Third, LME is able to incorporate categorical and continuous data into the same models, allowing us to account for the variance in performance due to the categorical variables (posture, word type, and word position), and the continuous variable (religiosity) in the same model without artificially segmenting the continuous variable (religiosity) into arbitrary categories (e.g., using a median split). Finally, the treatment of response accuracy as a dependent variable is challenging for ANOVA since accuracy is not normally distributed and therefore almost always incurs a violation of sphericity/homogeneity. To address this, LME can be used with the logistic function allowing us to use the same model selection procedure for reaction time (i.e., continuous) and accuracy (i.e., binomial) data.

Accuracy and RT data were analyzed using linear mixed effects models and a model selection procedure in *R* with the *lme4()* package (Bates et al., 2015). For the response latency analysis, only correct trials were used. Further, in line with Meier et al. (2007) we discarded trials that were faster than 300 ms and slower than 3,000 ms, and log transformed the remaining response latency data to better meet the assumption of normality. Importantly, such transformations are effective at normalizing positively skewed RT data (Marmolejo-Ramos et al., 2015). All models included random intercepts for each subject and though there are a small number of abstract words used in this study we included random intercepts for stimulus as well. For each fixed effect (i.e., independent variable), we compared models of increasingly complexity, starting with the most theoretically uninteresting variables (i.e., variables that should replicate patterns from previous research) and proceeded to include more theoretically interesting variables, for the purposes of the present study, in a step-wise manner. In all cases, participant intercept was included as a random effect.

For the model comparison procedure for the IAT, we compared three models:

- A model with word congruence (congruent vs. incongruent) as a fixed effect factor as this is the basic IAT effect that we expected to robustly replicate (i.e., DV ~ Word Congruence + (1 |Participant) + (1|Stimulus));
- A model with the Congruence X Posture (sitting, kneeling) interaction, to examine bodily grounding (i.e., DV ~ Word Congruence * Posture + (1 |Participant) + (1|Stimulus));
- A model that included the Congruence X Posture X Religiosity (continuous) interaction to explore whether lower order interactions are modulated by a participant’s religious experiences (i.e., DV ~ Word Congruence * Posture * Religiosity + (1 |Participant) + (1|Stimulus)).

We compared the fit of these models using likelihood comparison with the *anova()* function in R and selected the model with the lowest Akaike Information Criterion (AIC) value. For any complex model deemed significant, we visualized the highest order interaction with confidence intervals around the estimated marginal means from the fitted model. Rather than reporting a host of *post hoc* tests^2^, our interpretations were driven by theoretically interesting patterns within the estimated means (we interpret non-overlapping confidence intervals to suggest a theoretically interesting differences). Visualization of interactions, estimated marginal means, and confidence intervals were calculated using the *emmeans()* package (Lenth, 2020).

For the CAT, the four models we compared were:

- A model with word position (top vs. bottom) as this has already been shown to affect categorization performance (i.e., DV ~ Word Position + (1 |Participant) + (1|Stimulus));
- A model with the Word Position X Word Type (god, devil, power, no, sea, sky) interaction, as the different classes of words may lead to different effects of spatial position (i.e., DV ~ Word Position * Word Type + (1 |Participant) + (1|Stimulus));
- A model with the Word Position X Word Type X Posture (sitting vs. kneeling) as this is our theoretically interesting novel manipulation that seeks to explore bodily grounding (i.e., DV ~ Word Position * Word Type * Posture + (1 |Participant) + (1|Stimulus));
- A model with the Word Position X Word Type X Posture X Religiosity (continuous) interaction, as we seek to explore whether lower order interactions are modulated by a participant’s religious experiences (i.e., DV ~ Word Position * Word Type * Posture * Religiosity + (1 |Participant) + (1|Stimulus)).

Again, for the best fitting complex model (based on AIC), we visualized the highest order interaction with confidence intervals around the estimated means.

Finally, we report the marginal *R^2^* of the best fitting complex models using the *MuMIn()* package (Barton, 2020).

## Results

### Implicit association task

**Figure 2:**
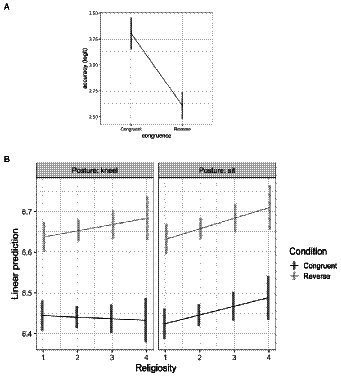
**(A)** Response accuracy (logit) as a function of congruence. **(B)** Log response latency as a function of congruence, posture, and religiosity. Bars represent confidence intervals on the estimated marginal means.

For the response accuracy data, none of the more complex models were a better fit than the simplest model, which included an effect of congruence (*b* = −0.7, SE = 0.07, *z* = −9.4, *p* < 0.001). This result was due to larger accuracy in the congruent condition than the reverse condition, replicating the basic IAT effect here with GOD/UP and DEVIL/DOWN. Figure 2A. Note, this indicates that religiosity and posture had no discernable effect on response accuracy.

For response latency data, the most complex model with the Congruence X Posture X Religiosity interaction (*npar* = 11, AIC = 5779.1, X^2^ = 28.7, *p* < 0.0001, *R^2^_m_* = 0.11) was a better fitting model than the next most complex model (*npar* = 7, AIC = 5799.8). Figure 2B. The confidence intervals suggest that, overall, participants were faster at categorizing congruent words, again replicating the basic IAT effect. However, this difference increased with greater religiosity when participants were kneeling, though the difference remains relatively constant while participants were sitting; that is, the IAT effect is more pronounced in more religious participants when they are kneeling.

### Categorization task

**Figure 3:**
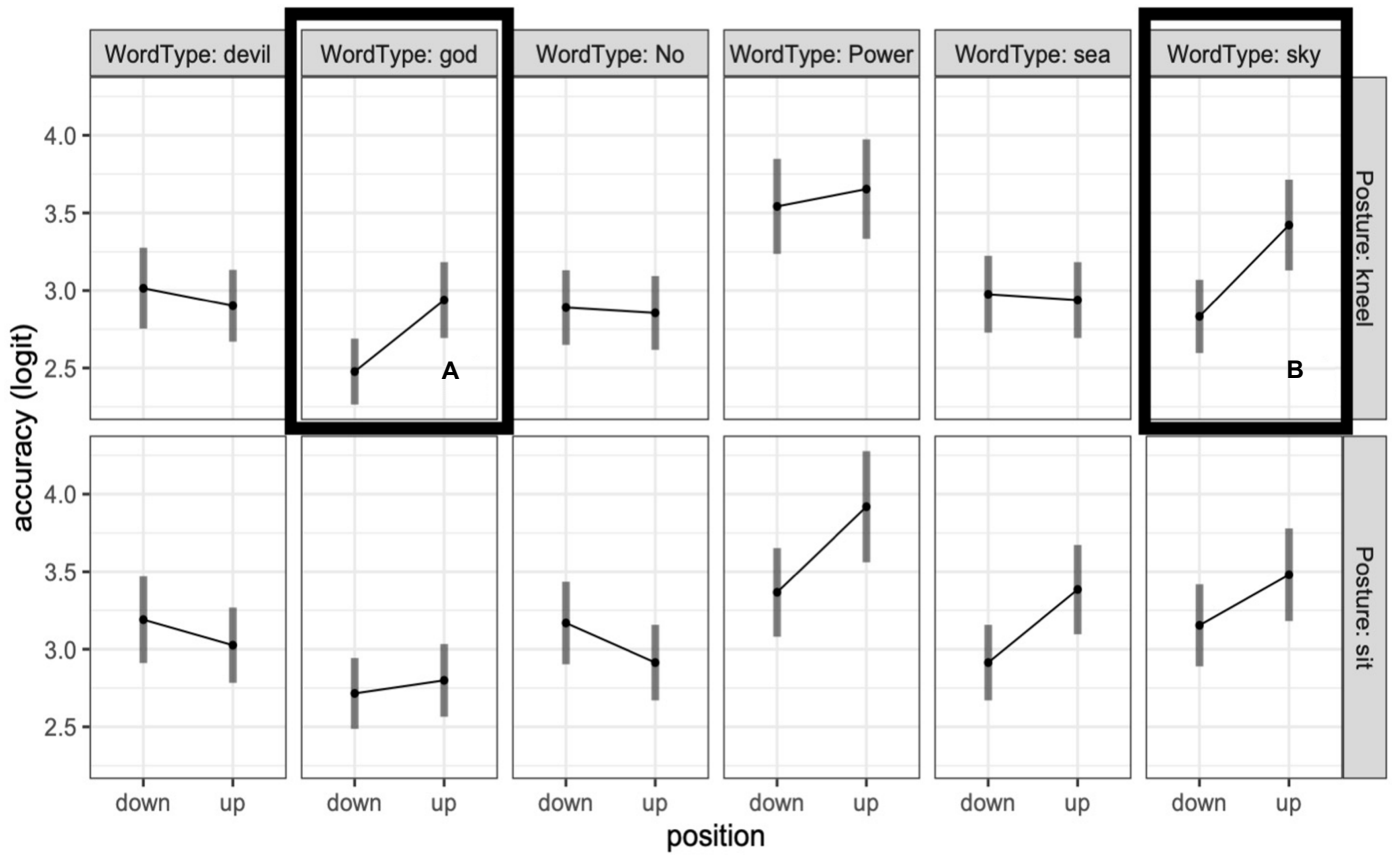
Categorization accuracy as a function of word type, posture, and word position. Error bars represent confidence intervals on the estimated marginal means from the LME model.

For response accuracy, a model including the two-way Word Position X Word Type interaction (*npar* = 14, AIC = 14,352, *X*^2^ = 25.4, *p* = 0.005, *R*^2^_m_ = 0.02) was a better fit than the simple model (*npar* = 4, AIC = 14,357). The two-way interaction is visualized in Figure 3.

This interaction appears to arise because there are trends for higher accuracy in the GOD-related, SKY-related, and POWER-related words when they are presented in the up position, with smaller differences between up and down position for the other words. However, the non-overlap in confidence intervals points to the overall higher accuracy in the POWER-related words as a primary driver of this interaction.

**Figure 4:**
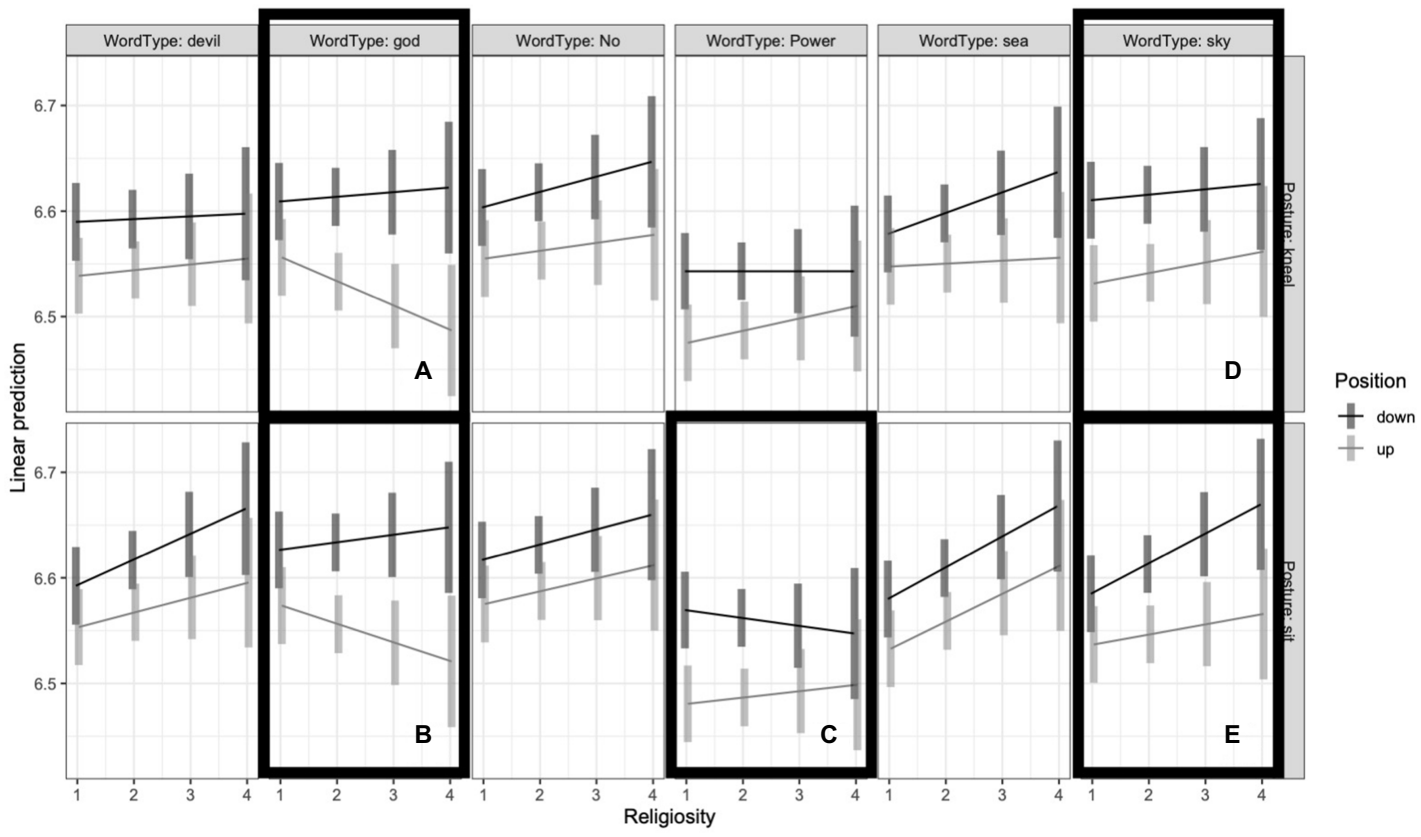
Log response latency as a function of word type, position, posture, and religiosity. Bars represent confidence intervals on the estimated marginal means.

For response latencies, the most complex model with the Word Position X Word Type X Posture X Religiosity interaction (*npar* = 51, AIC = 3638.9, *X*^2^ = 92.6, *p* < 0.0001, *R*^2^_m_ = 0.025) was a better fitting model than the next most complex model (*npar* = 27, AIC = 3683.5). The four-way interaction is visualized in Figure 4.

In the response latency data, the non-overlapping confidence intervals suggest that this interaction is being driven by the interaction between religiosity and position for the GOD-related words: Specifically, although in general participants were faster to categorize GOD-related words in the up position, this tendency increased as religiosity increased. (Note: this is especially apparent with the non-overlapping confidence interval when religiosity = 3 for GOD-related words while participants are kneeling). This difference does not seem to interact with posture.

## Discussion

According to the grounded cognition framework (Barsalou, 1999, 2008), sensorimotor information is at least partially constitutive of knowledge. Inspired by the CDZ framework (Meyer and Damasio, 2009), a neurocognitive model we take to exemplify the grounded cognition approach, we investigated the role of specific sensorimotor information that might ground abstract GOD-related and DEVIL-related concepts. Specifically, we hypothesized that action-oriented and visuospatial elements partially ground the representation of these concepts. To test for the functional role of such information, we manipulated the posture of participants and the visual location of where words were presented on screen across two different word categorization tasks. In summary, our results suggest the action of kneeling activates relevant sensorimotor information by which abstract GOD-related concepts are grounded. These results also suggest that visuospatial information is a part of GOD-related concepts, replicating the effect reported by Meier et al., 2007 (replicated also in Meier et al., 2021). Finally, we showed that aspects of sensorimotor grounding were predicted by a person’s religiosity, an individual differences variable we take to reflect experience with bodily and visual depictions of religious concepts. We discuss each of these results below.

First, we replicated the finding in the CAT that GOD-related words were categorized more quickly when they were presented at the top of the screen (Meier et al., 2007, 2021). This suggests that ‘up-ness’ is a visuospatial feature that influences categorization of GOD-related words. According to the CDZ framework, categorization is facilitated because the visuospatial information, a key source of grounding, is active during categorization. We extend this finding with two additional results that are important. First, for response latencies, more religious participants showed a greater GOD-UP advantage. This suggests that religious experience tends to strengthen the grounding of GOD-related concepts in the visuospatial domain (there are no detectable effects from kneeling on response latency). Importantly, the results of the CAT are complimented by the results in the IAT. First, in the IAT we replicated the categorization accuracy advantage for GOD-UP pairings (Meier et al., 2007). Importantly, however, our RT results extend this finding by showing that this advantage is larger for more religious participants when they are kneeling. Again, this suggests that bodily information and visuospatial information, when both active, contribute to categorization. Overall, the results of the present study suggest that bodily information, visuospatial information, and deeper experience with religious depictions and texts result in changes in the strength of sensorimotor grounding of abstract concepts.

**Figure 5:**
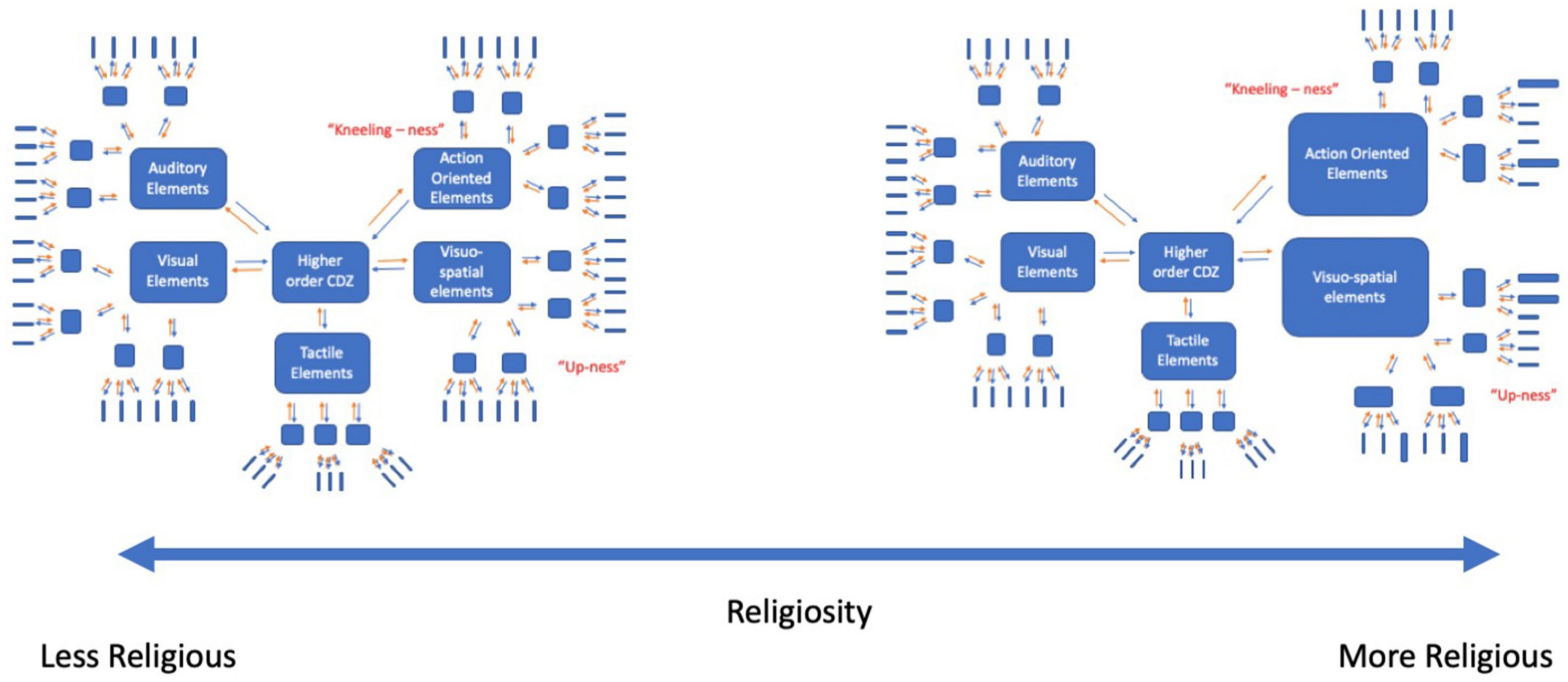
Schematic representation of the changes in the sensorimotor aspects of knowledge representation of GOD-related concepts as religiosity increases. Larger size of a subset of components in the Action-Oriented and Sensorimotor Elements denotes increase in relative importance or weighting of those components to conceptual representation as religiosity increases.

Figure 5 provides a conceptual summary of our interpretation of the results from a grounded cognition perspective, in which the ‘up-ness’ and ‘kneeling-ness’ of hypothesized representations of GOD-related concepts is modulated by a person’s over all religiosity. That is, according to models like the CDZ, concepts are partially grounded in the activation of sensorimotor information across different modalities (e.g., visuospatial, bodily, auditory, etc.). We take the aggregate religiosity scores of the present study to index experience with religious symbols and ritual. Thus, in more religious participants the action of kneeling is often associated with engagement with these kinds of concepts (e.g., when adopting the supplicative kneeling posture of prayer). In accordance with the CDZ model, we suggest that engaging in the kneeling posture results in activation of action-oriented sensorimotor information that supports the representation of GOD-related concepts and that this information is more relevant for highly religious people. Since activation in part of the representation can induce activation in the rest of the system, we suggest that the activity in these action-oriented regions work together with the associated visuospatial information of the representation, resulting in faster and more accurate categorization of the words overall.

The control word categories were chosen because they shared visuospatial features with the GOD-related and DEVIL-related concepts, specifically an appeal to a power hierarchy or literal location in space. Within these data, there are a number of additional trends in our results that we can speculate about. For instance, an unexpected finding was that, when categorizing POWER-related words, seated participants were faster to respond to these words in the up position if they scored lower in religiosity. We did not predict this, but can offer post-hoc speculations about why this is the case. For instance, it may be the case that people who are less religious are more sensitive to power hierarchies in the secular world, whereas religious participants have a learned a power dynamic that situates their deity as the highest power. This might result in an almost paradoxical effect, whereby concepts of social POWER are more visuospatially grounded in secular participants. While this is merely speculation, future research should explore this intriguing possibility.

There are several potential limitations to the present study. First, the surveys that we used and the GOD-related and DEVIL-related concepts selected from Meier et al. (2007) were predominantly driven to investigate religious people of Christian faiths and thus capture the visual and bodily experience of people of that religious group. Two of the subscales, the Religious Commitment Inventory and the Nearness to God Inventory have been used in secular populations (i.e., public universities) with success; however, these studies only minimally sample those of non-Christian faiths, and as such we cannot generalize our results to religious people outside of this faith group (Worthington et al., 2003). Thus, the generalizability of the results is clearly limited by the specific religious concepts we used and the religious experiences of our participants. Second, all of our interesting effects occurred with categories that were associated with UP (i.e., GOD, POWER), but not the comparable categories that we anticipated would be associated with DOWN (i.e., DEVIL, POWERLESS). Previous research has also found effects primarily with the categories associated with UP (Meier et al., 2007). Why these findings are the case remain unclear, though the obvious conclusion is that UP is more obviously visuospatially experienced than DOWN, since objects like the ocean and people with lower social power are more likely experienced not as below us *per se*, but rather at ground level where we have most of our experiences. This might reduce the usefulness of grounding concepts like DEVIL, OCEAN, and POWERLESS in visuospatial ‘down-ness’. Relatedly, another possibility is that grounding of these concepts relies heavily on interoceptive experience in addition to sensorimotor experiences of space and body posture. Indeed, interoception may be a primary modality for grounding abstract concepts (e.g., Kousta et al., 2011; Duris et al., 2017), and thus faith concepts like DEVIL might be grounded more by affective information like other affectively loaded words (e.g., DEATH is understood spatially with up vs. down metaphors in different languages; see Gathigia et al., 2018). Future research should investigate the unique, weighted contributions of visuospatial, bodily, and interoceptive information in conceptual grounding (e.g., Lynott et al., 2020).

Third, our interpretations are primarily based on convergent reaction time evidence from two different tasks. While we argue that these tasks provide convergent evidence regarding categorization processes, the differences in task structure likely probe these categorization processes in different ways. For instance, the CAT requires simply identifying and categorizing words as they appear on the screen, whereas the IAT requires pre-activating associated conceptual information before the presentation of each word (and then categorizing them based on the activated associated information). Thus, the tasks vary to the extent they pre-activate information. However, our interpretations are based on models like the CDZ, where both ‘bottom up’ and ‘top down’ activation iteratively constitute categorization. In this way, the CAT and the IAT might be argued to probe these unique processes, respectively.

Finally, our results are readily interpreted in terms of the CDZ framework but we cannot rule out the possibility that kneeling and/or visuospatial position is merely *associated* with, rather than constitutive of, a representation of GOD-related concepts (i.e., they do not allow us to identify the format of representations; see Mahon and Hickok, 2016, for discussion). However, the effects of the bodily posture and visuospatial manipulations used in the present study suggest at least a *functional* role of visuospatial and bodily information, because these manipulations are not directly related to the task goals of categorization (i.e., they are task-irrelevant; Ostarek and Bottini, 2021). More direct manipulation of sensorimotor information is needed (e.g., *via* transcranial magnetic stimulation or the manipulation of bodily experience) to further lend support for our conclusions and future research should investigate this. As discussed, previous direct manipulation of motor cortices can facilitate lexical decisions on motor related words (Pulvermüller et al., 2005), providing some evidence of a direct, functional role of sensorimotor information in the representation of knowledge. Thus, at the very least, our experimental manipulations remain suggestive of a functional role (if not a fully constitutive role) of visuospatial and bodily information in categorization.

In conclusion, accounting for the representation of abstract concepts is one of the most pressing challenges for grounded cognitive approaches to knowledge. Our results suggest that not only are sensorimotor features associated with the experience of abstract concepts, but action-oriented and visuospatial experiences play a functional role in the representational structure that supports abstract concepts.

## Data availability statement

The datasets presented in this study can be found in online repositories. The names of the repository/repositories and accession number (s) can be found at: https://osf.io/qfp8d.

## Ethics statement

The studies involving human participants were reviewed and approved by Research Ethics Board University of Northern British Columbia. The patients/participants provided their written informed consent to participate in this study.

## Author contributions

SM, BD, AD, PS, and HM were involved in the conception of the project, the generation of research methods, and the preparation of the manuscript. SM was also involved in data collection. HM was additionally involved in guiding the questionnaire formulation, and data analysis. All authors contributed to the article and approved the submitted version.

## Funding

Support was provided by an NSERC Discovery Grant to HEM.

## Conflict of interest

The authors declare that the research was conducted in the absence of any commercial or financial relationships that could be construed as a potential conflict of interest.

## Publisher’s note

All claims expressed in this article are solely those of the authors and do not necessarily represent those of their affiliated organizations, or those of the publisher, the editors and the reviewers. Any product that may be evaluated in this article, or claim that may be made by its manufacturer, is not guaranteed or endorsed by the publisher.

## Footnotes

^1^We note that there has been much criticism of the embodied account of abstract concepts (e.g., Pecher, 2018), and criticisms are bolstered by numerous failed replications of social-priming effects that are often described as ‘embodied’ from social psychology. However, larger-scale direct replications have shown that the cognitive GOD/UP association is replicable in the implicit associations task (Meier and Fetterman, 2022) and in categorization tasks (Meier and Fetterman, 2022) we used here.

^2^Note that code for post hoc tests is available in the analysis script at https://osf.io/qfp8d/
